# Scrapping the Trained Nurse

**Published:** 1920-03-13

**Authors:** 


### SCRAPPING THE TRAINED NURSE.

Readers of The Hospital may remember a short article in a recent issue (February 14) advocating the training of probationers in Metropolitan and other standard hospitals for ultimate employment as health visitors; and complaining that outside women are being trained in outside institutions to fulfil functions for which the trained nurse is a vastly superior candidate. I wrote in uncompromising terms, for I hold the view that for fitness in the great work of health reconstruction the trained nurse is without a peer. This, in the first place. Secondly, I hold the view that the nation and the voluntary hospital both owe an unpayable debt to the trained nurse.

The decision of the British people to reconstruct the nation's health opens up a new field of employment for women. Health visitors, much better paid than hospital nurses, will be appointed all over England to fulfil responsible duties; but I find an iron gate, locked and barred, with the trained nurse on the wrong side.

Realising that I had been beating the air, I sent this article to Dr. Addison, and frankly asked him where he stood in the matter. Dr. Addison sent me, in reply, a quantity of very interesting literature, which documents, as he says, "show the attitude of the Ministry with regard to the work and training of health visitors."

"I am to point out," writes the Minister's secretary, "that the special curriculum which has now been prescribed for the training of health visitors by the Board of Education in consultation with this Ministry could not be provided at a hospital!"

Attend lectures at the Polytechnic! Listen to University men on physiology, hygiene, and kindred topics. Study the text-books, and pass examinations. Thus you become a health visitor.

Mark you, I should agree if it were laid down that jazzing instruction "could not be provided at a hospital," or that bombing practice for soldiers would there be out of place. The hospital is not an omnibus. What I expected the Minister to state was that adequate training for a health visitor could not be provided except at a hospital. This, I regret to observe, he did not say. Dr. Addison is out in quest of students of institutions connected with Universities—schoolwomen, in fact. If the trained nurse would enter she must attend such an institution for a year. She must give up her employment, go into apartments, and become a speculative student If she cannot go she is turned down.

The Hospital Attitude.

I submitted Dr. Addison's letter and the printed curriculum of study to the matron of a large and up-to-date hospital. This lady possesses exceptional attainments and wide experience. She read these with amazement, and informed me that as a probationer at the London Hospital she had been thoroughly instructed in practically the entire programme. If in her own hospital she had the opportunity, she assured me that she would snap at the privilege of being permitted to provide the necessary equipment, and in participating in the grants awarded by the Ministry of Health for training students. What say other matrons?

The hospital nurse has been flattered, haloed, and even registered. In the war she worked with the zeal of an enthusiast, and shared the common fate and the common grave of the men-at-arms. Now, when reconstruction comes along, she is as a tale that is told.

IERNE.

[We print "Ierne's" jeremiad, but in bare justice to Dr. Addison and his Department we feel obliged to add that his letter to "Ierne" shows that, whereas the curriculum of instruction in health-visiting duties for ordinary candidates lasts two years, trained nurses are enabled to complete it in one year. It is reasonable to suppose that in this one year the trained nurse will be instructed in various matters outside the scope of her instruction as a nurse. If, then, the second year allotted to non-trained nurse candidates is devoted to instruction in that to which the trained nurse has already devoted three years, the latter will obviously start in the service with marked advantages of efficiency, which should soon lead her to the most responsible and best-paid positions.—Ed. The Hospital.]

